# Group Practice Performance in the Second Year of Medicare’s Merit-Based Incentive Payment System

**DOI:** 10.1001/jamanetworkopen.2021.28267

**Published:** 2021-10-05

**Authors:** Joshua M. Liao, Lingmei Zhou, Amol S. Navathe

**Affiliations:** 1 Department of Medicine, University of Washington School of Medicine, Seattle; 2 Value and Systems Science Lab, University of Washington School of Medicine, Seattle; 3 Leonard Davis Institute of Health Economics, University of Pennsylvania, Philadelphia; 4 Corporal Michael J. Crescenz VA Medical Center, Philadelphia, Pennsylvania; 5 Department of Medical Ethics and Health Policy, Perelman School of Medicine, University of Pennsylvania, Philadelphia

## Abstract

This cross-sectional study describes performance among group practices in the second year of the Medicare Merit-Based Incentive Payment System (MIPS) program.

## Introduction

The Merit-Based Incentive Payment System (MIPS) program seeks to reward clinicians for practicing high-value care in the following 4 domains: quality, resource use, advancing care information, and improvement activities.^1,2^ A composite performance score is calculated and compared with a threshold score to positively or negatively adjust clinicians’ Medicare fee-for-service payment rates by up to 7% in 2021 and 9% in subsequent years.

In 2020, nearly 900 000 clinicians nationwide received MIPS payment adjustments based on 2018 performance. Because nearly 80% were part of group practices, and practices were evaluated using different criteria than individuals, understanding group performance is central to understanding overall MIPS performance. In 2019, the first year of MIPS adjustments, the composite score threshold was very lenient: Groups avoided penalties simply by reporting data, and the vast majority of groups were deemed exceptional performers. However, little is known about how groups fared as MIPS rules for 2020 adjustments became more stringent. The objective of this study was to describe performance among group practices in the second year of MIPS.

## Methods

We conducted this cross-sectional study among group practices between March and July 2021. Our unit of analysis was the group practice. We linked 2018 MIPS group practice data with Physician Compare data to calculate practice size, urban or rural status, scope (single vs multispecialty), and practice Medicare population size and case mix (beneficiaries’ average Hierarchical Condition Category scores).^2,3,4^ We used county-level data to define characteristics of practices’ surrounding communities, including health care spending (Medicare per-beneficiary reimbursement), educational attainment (proportion of individuals with some college education), income (median household), and severe housing cost burden (proportion of individuals spending >50% of their income on housing).^5^ Study data were publicly available, and the University of Washington institutional review board waived approval per institutional policy. We followed the Strengthening the Reporting of Observational Studies in Epidemiology (STROBE) reporting guideline where appropriate.

Per Medicare rules, we categorized MIPS participants by composite score into negative (<15), neutral (15), positive (>15 and <70), and exceptional (≥70) performers. We conducted pairwise comparisons between exceptional and other performance categories using χ^2^ tests for categorical variables and Kruskal-Wallis tests for continuous variables. Statistical tests were 2-tailed and significant at α = .05. Analyses were performed in SAS, version 9.4 (SAS Institute Inc).

## Results

Our sample consisted of 17 201 group practices representing 700 706 clinicians. Of the 17 201 group practices, 66.2% were exceptional performers, 33.3% were positive performers, and very few were neutral (0.1%) or negative (0.5%) performers. The mean (SD) MIPS composite score was 76.0 (27.5), and the median (IQR) score was 89 (52.7-100.0) (Figure).

**Figure.**
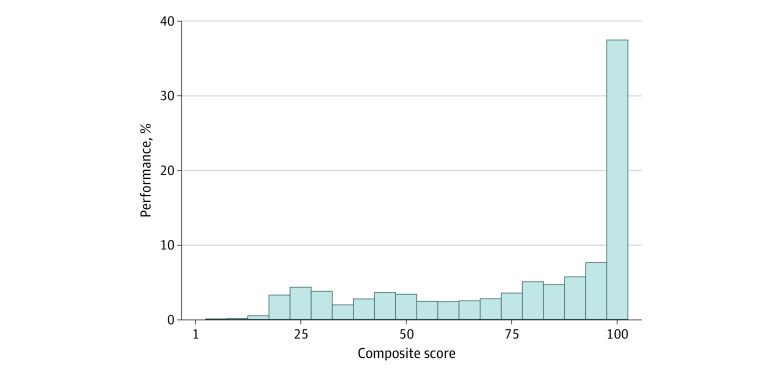
2018 Merit-Based Incentive Payment System Performance Distribution Among 17 201 Group Practices

Across groups, 16 115 (93.7%) matched to Physician Compare and county-level data (Table). Most of these were small practices (11 083 [68.8%]) located in urban areas (13 638 [84.6%]) with college-educated individuals (median [IQR] of 65.7% [59.4%-71.3%] for residents with some college) without housing cost burden (median [IQR] of 14.4% [12.3%-17.4%] of residents experiencing severe housing cost burden). Most groups were multispecialty (11 391 [70.7%]).

**Table.** Characteristics Associated With 2018 Merit-Based Incentive Payment System Performance Among Group Practices

Characteristic	Overall (N = 16 115 [100%])	Performer Category			
		Negative (n = 70 [0.5%])	Neutral (n = 13 [0.1%])	Positive (n = 5271 [33.3%])	Exceptional (n = 10 761 [66.2%])
**Practice characteristics**					
Size, No. (%)^a^					
Small	11 083 (68.8)	45 (64.3)	10 (76.9)	3469 (65.8)^b^	7559 (70.2)
Other	5032 (31.2)	25 (35.7)	3 (23.1)	1802 (34.2)	3202 (29.8)
Urban or rural, No. (%)					
Urban	13 638 (84.6)	59 (84.3)	12 (92.3)	4381 (83.1)^b^	9186 (85.4)
Rural	2477 (15.4)	11 (15.7)	1 (7.7)	890 (16.9)	1575 (14.6)
Census region, No. (%)					
Midwest	3222 (20.0)	15 (21.4)	2 (15.4)	1070 (20.3)^b^	2135 (19.8)
Northeast	3025 (18.8)	15 (21.4)	2 (15.4)	1052 (20.0)	1956 (18.2)
South	6676 (41.4)	20 (28.6)	3 (23.1)	2173 (41.2)	4480 (41.6)
West	3192 (19.8)	20 (28.6)	6 (46.2)	976 (18.5)	2190 (20.4)
Scope, No. (%)					
Single specialty	4724 (29.3)	21 (30.0)	4 (30.8)	1538 (29.2)	3161 (29.4)
Multispecialty	11 391 (70.7)	49 (70.0)	9 (69.2)	3733 (70.8)	7600 (70.6)
Patient population case mix, median (IQR) (n = 16 102)^c^	1.5 (1.2-1.9)	1.7 (1.3-1.9)	1.4 (1.2-1.9)	1.6 (1.3-2.0)^b^	1.5 (1.2-1.9)
Patient population size, median (IQR), No. (n = 16 102)	4250 (1631-12 541)	2040 (841-10 184)^b^	1768 (904-5037)	4018 (1457-12 993)^b^	4335 (1735-12 350)
Proportion of Medicare/Medicaid dual-eligible patients, median (IQR), %	20.3 (11.3-31.9)	18.2 (12.6-36.7)	24.0 (17.8-29.9)	23.0 (13.5-35.2)^b^	19.1 (10.4-30.4)
Proportion of Black patients, median (IQR), %	4.8 (1.6-10.9)	5.7 (2.4-11.1)	6.1 (2.9-8.5)	5.2 (1.7-11.2)^b^	4.5 (1.5-10.7)
**Community characteristics (n = 16 114)**					
Health care spending, median (IQR), $^d^	10 240 (9330-11 530)	10 302 (9263-11 530)	10 327 (9943-11 077)	10 315 (9371-11 589)^b^	10 167 (9297-11 518)
Proportion with some college education, median % (IQR)	65.7 (59.4-71.3)	64.7 (59.4-70.2)	68.2 (60.5-69.8)	65.6 (59.3-70.8)^b^	65.7 (59.4-71.8)
Median household income, median (IQR), $	58 264 (50 292-68 957)	58 154 (51 093-65 586)	51 850 (51 207-60 270)	57 862 (49 993-69 222)	58 366 (50 400-68 864)
Proportion with severe housing cost burden, median (IQR), %	14.4 (12.3-17.4)	14.6 (11.2-17.2)	15.1 (13.5-20.1)	14.5 (12.1-17.4)	14.4 (12.3-17.4)

Abbreviation: IQR, interquartile range.

^a^ Dichotomized following Medicare rules as small (≤15 clinicians) vs other (≥16 clinicians).

^b^ *P* < .05; tests compare exceptional vs other performance groups.

^c^ Case mix indicates beneficiaries’ average Hierarchical Condition Category scores.^3^

^d^ Spending reflects price-, age-, sex-, and race-adjusted Medicare per-beneficiary spending.

Several characteristics varied by MIPS performance (Table). Compared with the 70 negative performers, the 10 761 exceptional performers had larger patient populations, with a median [IQR] of 4335 [1735-12 350] vs 2040 [841-10 184] patients. Compared with 5271 positive performers, more exceptional performers were small practices (7559 of 10 761 [70.2%] vs 3469 of 5271 [65.8%]). Exceptional performers also had lower proportions of Medicare/Medicaid dual-eligible patients (median [IQR] of 19.1% [10.4%-30.4%] vs 23.0% [13.5%-35.2%]) and Black patients (median [IQR] of 4.5% [1.5%-10.7%] vs 5.2% [1.7%-11.2%]) than positive performers. There were small differences in urban status, regional health care spending, and community-level educational attainment between positive and exceptional performers.

## Discussion

The findings of this cross-sectional study suggest that despite more stringent composite score thresholds for 2020 payment adjustments, most practices achieved exceptional performance. Similar to the preceding year, performance varied by practice factors.^6^ Study limitations included unavailable payment adjustment information, the inability to infer causality about practice factors associated with MIPS performance, exclusion of the small proportion of clinicians (21%) participating in MIPS outside of group practices, and use of community-level variables. Nonetheless, performance thresholds could be revised to avoid designating most practices as exceptional, thereby diluting its meaning and policy impact. Policy makers could also consider practice factors to make MIPS scoring more equitable.
